# Nanotechnology-based combinatorial phototherapy for enhanced cancer treatment

**DOI:** 10.1039/d1ra09067d

**Published:** 2022-04-04

**Authors:** Han Chen, Peter Timashev, Yuanyuan Zhang, Xiangdong Xue, Xing-Jie Liang

**Affiliations:** School of Pharmacy, Pharm-X Center, Shanghai Jiao Tong Univeristy Shanghai 200240 China xuexd@sjtu.edu.cn; Laboratory of Clinical Smart Nanotechnologies, Institute for Regenerative Medicine, Sechenov University Moscow 119991 Russia; CAS Center for Excellence in Nanoscience, CAS Key Laboratory for Biomedical Effects of Nanomaterials and Nanosafety, Chinese Academy of Sciences, National Center for Nanoscience and Technology of China Beijing 100190 China liangxj@nanoctr.cn

## Abstract

Nanotechnology-based phototherapy has attracted enormous attention to cancer treatment owning to its non-invasiveness, high controllability and accuracy. Given the fast development of anti-tumor strategies, we summarize various examples of multifunctional nanosystems to highlight the recent advances in nanotechnology-based combinatorial phototherapy towards improving cancer treatment. The limitations of the monotherapeutic approach and the superiority of the photo-involved combinatorial strategies are discussed in each part. The future breakthroughs and clinical perspectives of combinatorial phototherapy are also outlooked. Our perspectives may inspire researchers to develop more effective phototherapy-based cancer-treating approaches.

## Abstract 2

In this review, we summarize the recently ingenious design of nanotechnology-based phototherapy combined with different therapeutic modalities, like chemotherapy, immunotherapy, radiotherapy, *etc.*, as well as its anti-cancer therapeutic benefits.

## Graphical abstract

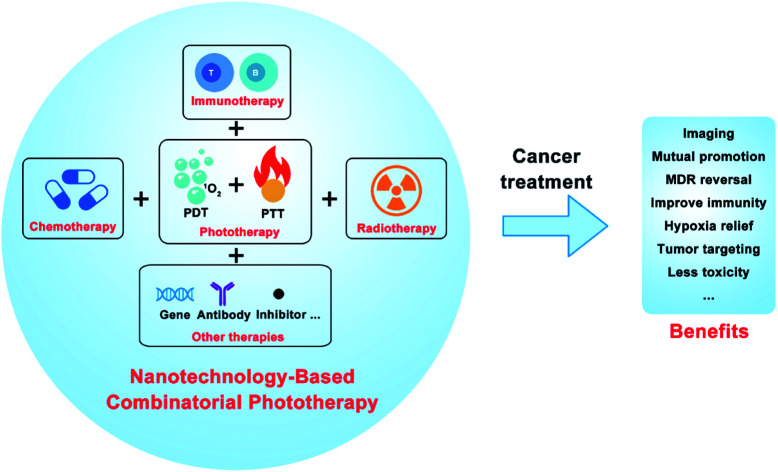

## 1. Introduction

Cancer is a life-threatening disease worldwide, commonly with a prevalence of >10 million mortalities annually.^1,2^ Various treatment approaches, such as surgery, chemotherapy,^3,4^ radiotherapy^5,6^ and immunotherapy,^7,8^ play an indispensable role in slowing or preventing tumor progression. Despite these approaches contributing excellent anticancer efficacy and subsequently prolonging the survival of patients, they still suffer from some shortcomings, such as serious side effects, drug resistance and inadequate response. In detail, the breadth of chemotherapeutic agents is vast, and adverse effects are common, unfortunately, due to the lack of selective action to tumor cells.^9,10^ Chemotherapy also encounters drug resistance developed by cancer cells, which severely reduces the intracellular concentration of therapeutics.^11,12^ For radiotherapy, the cancerous conditions, such as hypoxic tumor microenvironment (TME),^13^ acidic pH^14^ and dispersed tumor distribution,^15^ significantly limit its clinical applications. As for immunotherapy, low response rate and potential cause of autoimmune reaction remain major limitations.^16–19^ Phototherapies, including photodynamic therapy (PDT) and photothermal therapy (PTT), have attracted significant attention for highly efficient cancer treatment, owing to their non-invasive, controllable and accurate characteristics.^20–23^ PTT employs photothermal agents (PTAs) to transform the photonic energy to hyperthermia which enables ablation of a confined area irradiated by laser.^24^ However, PTT is limited by the inherent drawbacks of laser attenuation, nonuniform distribution of PTAs and undesirable phototoxicity on normal tissues.^25–27^ PDT is predicated on the generation of reactive oxygen species (ROS) to induce cytotoxic effects by photosensitizers, which directly damages protein, lipid, and DNA, resulting in cell death.^28,29^ PDT is recognized as a tumor-specific treatment paradigm, but the hypoxic TME significantly hinders its applications.^30,31^ What's more, the anti-tumor efficacy of phototherapy is far from ideal due to inadequate tumor accumulation of photosensitizer or PTAs, as well as the lack of strength to tumor metastasis.^32^ Therefore, it is necessary to develop new therapeutic modalities to improve the efficacy and safety of these anticancer approaches.

As discussed above, currently developed cancer-treating approaches, including chemotherapy, radiotherapy, immunotherapy and phototherapy, are barely satisfactory due to their unavoidable limitations. Moreover, cancer development involves complex biological processes; the highly malignant, aggressive, and heterogenetic nature of cancers^33^ would impede the anticancer effect when these therapeutic approaches work individually. Hence, a paradigm shift towards combing these therapeutic approaches could be a powerful solution to improve cancer treatments. With the fast development of nanotechnology, nanomedicine has become an emerging field that significantly improves the cancer-treating paradigm by intervening tumors at a molecular scale.^34,35^ Nanomedicine can integrate different therapeutic approaches into one single nanoparticle to realize a combinatorial or synergistic effect.^36,37^ Moreover, nanomedicine load and protect therapeutic agents in nanocarriers to improve their bioavailability and pharmacokinetics, and enable tumor accumulation by taking advantage of the enhanced permeability and retention (EPR) effect.^38,39^ Therefore, nanotechnology-based combinatorial therapies have great potential to remedy the shortcomings of the current anticancer strategies through non-overlapping cancer-killing mechanisms.^40,41^

Phototherapy shows considerable potential to complement chemotherapy,^42,43^ immunotherapy^44,45^ and radiotherapy,^46,47^ and exhibit potent synergistic anticancer efficacy. Furthermore, most organic photosensitizers or PTAs are naturally imaging agents which endow phototherapy concomitantly with versatile imaging functions, such as near-infrared fluorescent imaging (NIRFI),^42,48^ magnetic resonance imaging (MRI)^49,50^ and photoacoustic imaging (PAI).^51,52^ The integration of diagnostic and therapeutic modalities is a promising way to make cancer treatment more effective and precise.^41,53^ The diagnostic agents can vividly image the pathological characteristics of tumors, such as tumor development, malignancy and metastasis; meanwhile, the therapeutics can learn from these characteristics to adapt themselves for proper therapeutic strategies and individualized treatment. So far, numerous examples of phototherapy with imaging capacities have been developed with powerful diagnostic and therapeutic effects for cancer treatments.^54–56^

**Fig. 1:**
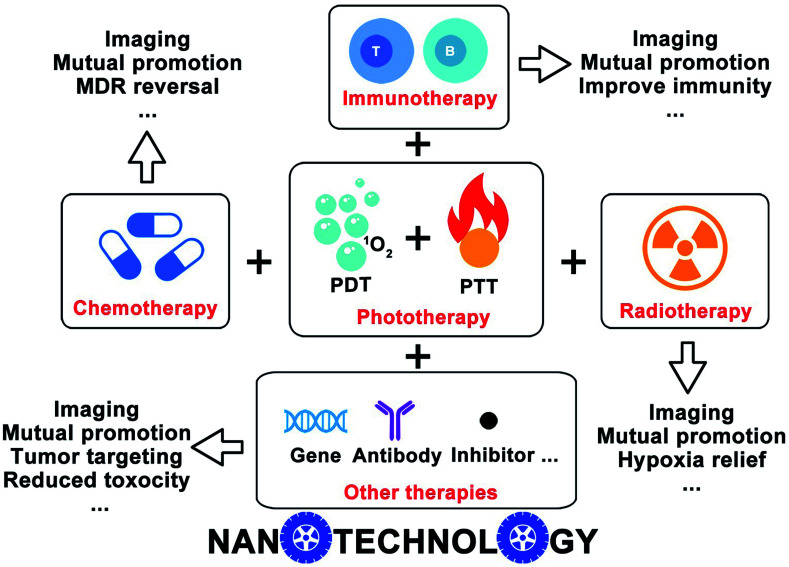
Schematic illustration of the nanotechnology-based combinatorial phototherapy.

In this review, we highlight the recently ingenious design of nanotechnology-based phototherapy combined with different therapeutic modalities, like chemotherapy, immunotherapy, radiotherapy, *etc.*, as well as the imaging capacities that come along with the phototherapy (Fig. 1). We discussed several examples and emphasized the benefits of combinatorial therapy against cancer. The clinical future of nanotechnology-based combinatorial phototherapy is also outlooked. We hope our overall envisions will inspire other researchers to develop more sophisticated nanomedicines which can extensively alleviate the side effects and improve the efficacy of the current therapeutic regimens.

## 2. Nanotechnology-based strategies for combinatorial phototherapy

Monotherapy remains challenging to suppress cancer progression due to the complexity of cancers. Combinatorial PTT/PDT and their integration with other therapeutic modalities could provide more opportunities to strengthen the advantages and avoid the disadvantages of the individual therapeutic approach. In the following sections, we mainly discussed the nanotechnology-based combinatorial therapies rather than a simple overlay of different therapeutic paradigms, as the therapeutics delivered by nanomedicine can reach the targeting locale simultaneously and their pharmacokinetics are uniformed. It is of paramount importance to integrate diagnostic and therapeutic modalities into one single nanoplatform to achieve real-time visualization of different pathological processes.^57^ Unlike the conventional nanomedicines that need extra steps to introduce imaging agents, most phototherapy-involved combinatorial strategies inherently have self-indicating features which would make the therapeutic regimens preciser and more effective.

### 2.1 Combinatorial PTT and PDT

**Fig. 2:**
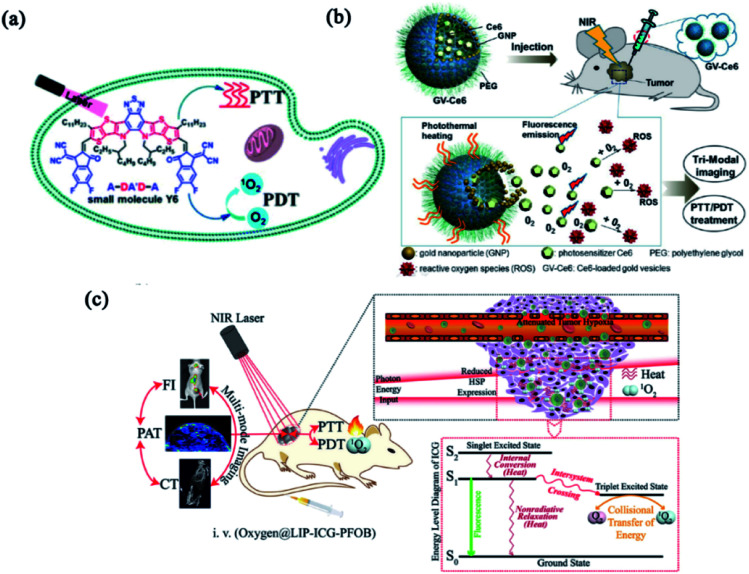
Combinatorial PTT and PDT. (a) Schematic illustration of Y6 NPs based on the A–DA′D–A fused-ring. Reproduced with permission from Yao *et al.* (2021).^59^ (b) Schematic illustration of GV-Ce6-based PTT/PDT treatment with tri-modal imaging. Reproduced with permission from Nie *et al.* (2013).^60^ (c) Schematic illustration of the functions and mechanisms of oxygen@LIP-ICG-PFOB. Reproduced with permission from Wang *et al.* (2018).^61^

Considering the mutual requirement between laser irradiation and similar functional mechanisms, combinatorial PTT and PDT were firstly proposed to treat port-wine stain by Nelson's group.^58^ The combination of PTT and PDT not only triggered the photothermally enhanced PDT, but also evoked a synergistic effect while decreasing the doses of therapeutics, thereby minimizing the dose-dependent side effects. Some photosensitizers, like porphin, phthalocyanine, porphyrins and other organic dyestuffs, were combined with different photo-conversion materials to build PTT/PDT combinatorial systems.^20^ Inspired by the push–pull electron effect between electron-donating units (D) and electron-withdrawing units (A), Yao group employed a small molecule photosensitizer (Y6) to build nanomedicine with combinatorial PTT and PDT for tumor treatment (Fig. 2a). Y6 was encapsulated in an amphiphilic polymer (DSPE-PEG2000) to form Y6 NPs with wide-spectral absorption (300 to 900 nm), high photothermal conversion efficiency (57%) and ROS production. The high photothermal conversion and superior photodynamic activity make Y6 NPs with great potential for combinatorial PTT and PDT against cancer.^59^ Nie and coworkers developed multifunctional Ce6-loaded plasmonic gold vesicles (GV-Ce6) for tri-modal fluorescence/photothermal/photoacoustic imaging-guided combinatorial PTT/PDT (Fig. 2b). The GV-Ce6 showed high Ce6 loading efficiency (∼18.4 wt%), multimodal imaging capacity and synergistic PTT/PDT effects by using single wavelength continuous-wave laser irradiation.^60^ Similarly, perfluorooctyl bromide (PFOB) & indocyanine green (ICG) co-loaded nanoliposomes (LIP-PFOB-ICG) realized computed tomography (CT) contrast imaging *in vivo*, providing better anatomical information of tumor in comparison to ICG enabled fluorescence and PAI (Fig. 2c).^61^ Synergistically integrating PTT and PDT is considered feasible to improve photo-induced therapeutic efficiency. Moreover, nanomedicines with self-indicating features are potential for clinical translation due to their real-time imaging in a non-invasive manner.

**Fig. 3:**
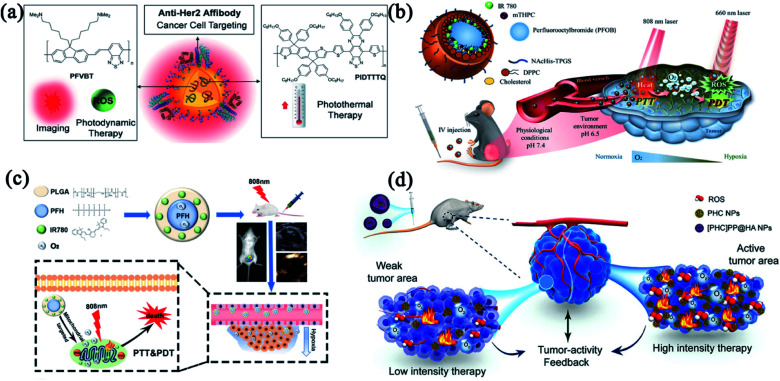
Combinatorial PTT and PDT with multiple functionalities. (a) Composition/structure and tumor-targeting capacity of anti-HER2-CPNs. Reproduced with permission from Liu *et al.* (2016).^107^ (b) Composition/structure and oxygen-delivering capacity of PFOB droplet NPs. Reproduced with permission from Chiu *et al.* (2020).^66^ (c) Schematic illustration of a mitochondria-targeted liquid fluorocarbon-based oxygen delivery system for imaging-guided PTT and PDT. Reproduced with permission from Niu *et al.* (2020).^67^ (d) The design of [PHC]PP@HA NPs for the combination of PTT and PDT. Reproduced with permission from Wu *et al.* (2020).^69^

Although enormous efforts have been devoted to designing excellent modalities that integrate PTT and PDT to considerably improve the therapeutic efficacy and offset the side effects, only limited success has been achieved due to the insufficient tumor accumulation of phototherapeutic agents and hypoxic TME.^62,63^ To reduce the off-target toxicity, different functional groups were introduced to endow nanomedicines with targeting features. Liu and co-workers designed a multifunctional polymeric nanoparticle (CPN) for image-guided combinatorial phototherapy with tumor targeting function (Fig. 3a). Two conjugated polymers, PFVBT with bright red fluorescence and efficient ROS production capability and PIDTTTQ with high photothermal conversion efficiency were encapsulated into lipid-polyethylene glycol (PEG) matrix to form a combinatorial PDT/PTT nanomedicine (CPN). The obtained CPNs showed a uniform size of 30 nm with a high ROS yield (60.4%) and effective photothermal conversion efficiency (47.6%). With the decoration of anti-HER2 antibody, the CPNs exhibited superior selectivity toward HER2 overexpressed SKBR-3 breast cancer cells.^64^ To ameliorate the hypoxic TME, combinatorial PDT/PTT nano-systems with oxygen-delivering capacity have gained great attention. Compared to the conventional exogenous oxygen delivery,^65^ nanoparticle-based delivery systems selectively accumulate in the tumor through an active or passive targeting pathway to trigger intratumoral reoxygenation. Chiu and co-workers fabricated a phospholipid membrane-enclosed PFOB droplets (PFOB@IMHNPs) with TME pH-responsiveness and excellent oxygen-carrying capability (Fig. 3b). The PFOB@IMHNPs can deliver oxygen, IR780 (for PTT/PDT) and *meta*-tetra(hydroxyphenyl)chlorin (*m*THPC for PDT) simultaneously, and accumulate in tumor site to improve the efficacy of combinatorial PDT/PTT.^66^ Niu and co-workers employed a mitochondria-targeting PFH NPs as an oxygen carrier which effectively alleviated the hypoxia in TME for improved tumor phototherapy (Fig. 3c). The resultant nanomedicine (IRP/O_2_ NP) was composed of perfluorohexane (PFH), IR780 and oxygen, which realized outstanding antitumor efficacy by an unprecedented design of tumor mitochondria targeting, oxygen delivery, combinatorial PDT/PTT and dual-imaging guidance.^67^ These fundings provide exciting strategies for oxygen delivery that can strengthen the hypoxia-hindered phototherapies.

Oxygen-delivery is a good solution to antagonize hypoxia-induced phototherapeutic resistance. However, oxygen release tends to be instantaneous and may lead to heterogenetic distribution of oxygen, leaving a potential mismatch between treatment intensity and tumor tissue activity.^68^ Wu and coworkers successfully designed an intelligent tumor-feedback nanomedicine ([PHC]PP@HA NPs, ∼140 nm) for combinatorial PTT/PDT (Fig. 3d). The [PHC]PP@HA NPs were constructed by encapsulating smaller PHC NPs (hemoglobin and chlorin e6 loaded in polydopamine) in a polymeric micelle and then capped with hyaluronic acid (HA) to realize tumor-targeting action. The [PHC]PP@HA NPs experienced size changes from 140 to 10 nm in acidic TME, thereby exhibiting deep tissue penetrability in tumor. The [PHC]PP@HA NPs can adjust the oxygen release and PHC destruction based on the feedback signal of tumor activity, and exhibit extremely high PTT/PDT synergistic effect against prostatic adenocarcinoma by taking advantage of the multifunctional building blocks.^69^

### 2.2 Combinatorial cancer treatments with phototherapy and chemotherapy

Although combinatorial PTT and PDT have been reported to improve therapeutic efficiency, limited tissue penetration of light still hinders their applications to the deep-bedded tumor.^23^ Combining phototherapy with other light-independent therapies can solve this problem. As a leading clinical treatment, chemotherapy employs cytotoxic agents to combat many cancer types at different stages. Chemotherapeutic agents, including cytotoxic antibiotics (*e.g.*, doxorubicin), alkylating agents (*e.g.*, cisplatin), anti-metabolites (*e.g.*, gemcitabine), anti-microtubule agents (*e.g.*, paclitaxel), and topoisomerase I inhibitors (*e.g.*, irinotecan), have been widely utilized in clinical practice.^70^ Despite the clinical effectiveness of chemotherapy preventing tumor development to some extent, the drug resistance and severe side effect are still non-negligible.^71^ To obtain better anticancer effect, several multifunctional nanomedicines that combine chemotherapy and phototherapy are developed by taking advantage of their mutual promotion interactions.

**Fig. 4:**
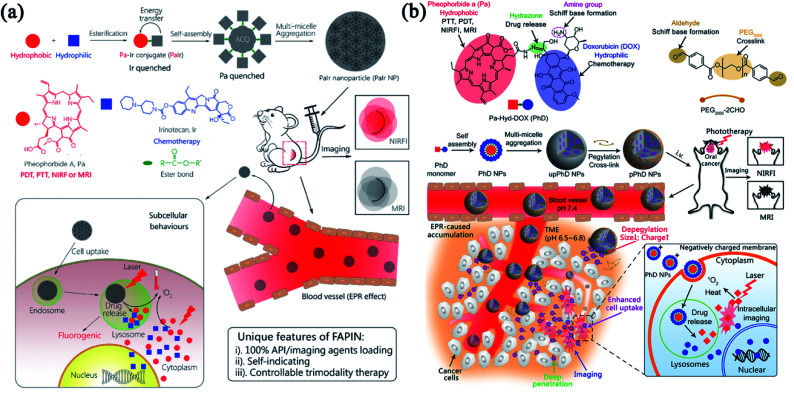
Combinatorial phototherapy and chemotherapy. (a) Schematic illustration of the self-indicating, fully active pharmaceutical ingredients nanoparticles (FAPIN). Adapted with permission from Xue *et al.* (2018).^80^ (b) The size/charge dual-transformable nanomedicine with combinatorial photo-/chemo-therapy against oral cancer. Reprinted with permission from Xue *et al.* (2018).^81^

On account of the abnormal tumor vasculature caused by EPR effect, the improved accumulation of nanoparticles is allowed at different solid tumors.^72–74^ However, low vascular density and hypoxic TME severely constrain the EPR effect during the drug delivery.^75,76^ Thus, the EPR effect alone is ineffective in spreading therapeutic agents throughout the tumor.^77^ PDT and PTT have been reported to permeabilize the tumor vasculature and facilitate intracellular translocation of anticancer drugs for enhanced chemotherapy with accurately controlled release property.^20^ Inspired by our previous work on small-molecule nanomedicines,^78,79^ we designed a self-indicating, fully active pharmaceutical ingredients nanoparticle (FAPIN or PaIr NP), which exhibited excellent dual-modal imaging capacities (MRI and NIRFI) and tri-modal therapeutic effects (PDT, PTT and chemotherapy).^80^ As shown in Fig. 4a, PaIr NPs were composed of two active pharmaceutical ingredients (APIs), pheophorbide A (Pa) and irinotecan (Ir), and exhibited 100% drug loading without adding additional excipients. In PaIr NPs, hydrophobic Pa contributed driving force for self-assembly, phototherapeutic effects, NIRFI and MRI; while Ir functioned as an anti-neoplastic drug that inhibited DNA topoisomerase II for chemotherapy. In addition, PaIr NPs exhibited self-indicating functions that can visualize the *in vivo* distribution and the light-triggered drug release in a real-time manner. With the potent synergism between phototherapy and chemotherapy, only 2 doses of PaIr NPs can cure 50% of mice with patient-derived xenograft (PDX) of glioblastoma.

As such, PaIr NPs were prepared with 100% APIs. However, the pharmacokinetics were compromised due to the bare drug surface and strong positive charge. Nanomedicine also faces many biological barriers when it circulates in the blood, infiltrates into tumor tissues and internalizes into tumor cells. To strengthen the FAPIN-like nanomedicine with robust stability and talent to overcome different biological barriers, we developed an “on-surface” crosslinked strategy^81^ that can tightly hold the FAPIN architecture and enable the drug release in the particular TME. As shown in Fig. 4b, we conjugate Pa and doxorubicin (DOX) through a hydrazone bond to form an amphiphilic drug–drug conjugate (PhD monomer) which can further assemble into PhD NPs as PaIr did. Taken further, we employed dialdehyde functionalized PEG2000 to crosslink the PhD NPs to form pPhD NPs *via* the formation of the Schiff base. pPhD NPs kept relatively large particles size and close-to-neutral surface charge which can escape the renal clearance to small particles and avoid opsonization of positively charged particles. In the slightly acidic TME, the cleavage of Schiff base peeled the PEG-crosslinker and released nanoparticles with a much smaller size (4 nm) to gain deeper tissue penetration. Meanwhile, the de-PEGylation can re-expose the amine groups on the particle surface and lead to the elevation of the surface charge. The strong positive charge extensively promoted the cellular internalization of small PhD NPs. The PhD NPs were transported into the lysosome, where the acidic pH can destroy the hydrazone bond to release Pa and DOX. Like in PaIr NPs, Pa exhibited excellent PDT, PTT and imaging functions (MRI and NIRFI). DOX exhibited a combinatorial chemotherapeutic effect with phototherapy. Due to the highly efficient delivery capabilities and the substantially combinatorial effect between phototherapy and chemotherapy, the pPhD NPs showed a 100% complete cure rate on subcutaneous and orthotopic oral cancer models. The pPhD NPs were also employed to reverse the drug resistance by inhibiting dual AKT/ERK pathways and efficiently ablate bladder cancer.^82^ The complex modification might lead to manufacturing complexities that impede their clinical translation.^83^ Hence, the FAPIN-like nanomedicine can significantly simplify nanoscale drug formulations, which may shed new light on large-scale production and clinical translation.

**Fig. 5:**
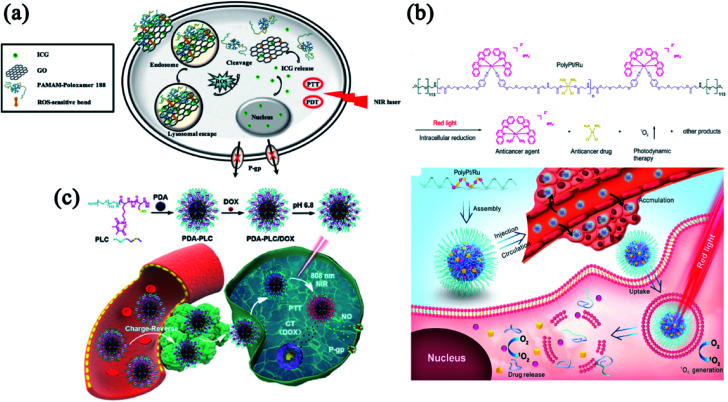
Combinatorial phototherapy and chemotherapy to combat drug resistance. (a) Composition/structure of ICG-loaded GPP and its biological performance. Reprinted with permission from Gao *et al.* (2019).^90^ (b) Schematic illustrating the proposed mechanism of Poly/Pt/Ru NPs. Reproduced with permission from Liang *et al.* (2020).^91^ (c) Schematic illustration of PDA-PLC/DOX NPs with enhanced PTT and NO-mediated gas therapy to overcome MDR. Reproduced with permission from Dong *et al.* (2021).^93^

Multidrug resistance (MDR) is considered a major challenge on cancer treatments.^84^ Increased drug efflux *via* P-glycoprotein (P-gp) or other similar efflux pump has been characterized as a major mechanism of drug resistance.^70,85–87^ One important strategy for overcoming drug resistance is fabricating phototherapy-involved multimodal nanomedicine to modulate the efflux mechanism.^88^ Phototherapy can mitigate the co-activation and compensation of molecular signaling pathways related to drug resistance when combined with chemotherapy and molecular targeted therapies.^89^ To overcome drug resistance, Gao and co-workers reported a ROS and light dual-sensitive nanohybrid constructed with diselenide cross-linked polyamidoamine-poloxamer 188 and graphene oxide with indocyanine green (ICG) as payloads (Fig. 5a). This nanohybrid enhanced the stability of ICG and exhibited ROS-sensitive drug-releasing behavior.^90^ ICG can accumulate in the nuclei of drug-resistant cancer cells and show anti-MDR properties by down-regulating the P-gp expression. This study provides a strategy for designing a nucleus-delivery nanocarrier with PDT and PTT effects that successfully overcome drug resistance. MDR also hinders the curative effects of cisplatin in clinical practice. To improve the therapeutic effect of cisplatin-mediated chemotherapy, we synthesized stimuli-responsive multi-metallic polymers (Poly/Pt/Ru) and assembled them into nanomedicine to alleviate cisplatin resistance (Fig. 5b).^91^ Upon laser irradiation, Poly/Pt/Ru nanoparticles generated ROS to induce polymer degradation and trigger the release of Ru(ii) to kill cancer cells. Meanwhile, the Pt(iv) in the polymer was reduced to cisplatin in the intracellular environment. The synergistic effect between PDT and chemotherapy can effectively inhibit drug-resistant tumors.

Bioactive gasotransmitter provides a novel strategy to maximize chemotherapeutic efficacy and realize MDR reversal.^92^ As a gasotransmitter, nitric oxide (NO) has been demonstrated to inhibit the P-gp expression. In Fig. 5c, Dong and co-workers developed a NIR/pH dual-sensitive charge-reversal polypeptide nanomedicine (PDA-PLC) to co-deliver NO donor and DOX.^93^ The distinctive charge-reversal capacity of PDA-PLC/DOX significantly facilitated cellular uptake of the payloads. In addition, the NIR-triggered NO release effectively inhibited the P-gp expression and subsequently suppressed the MDR tumor *via* the combinatorial therapies of PTT, gas therapy and chemotherapy. These exciting results of MDR reversal would inspire the development of more effective chemotherapy in clinical practice.

### 2.3 Combination of phototherapy with radiotherapy

Phototherapy alone sometimes fails to arrest cancers, especially those with deep-located tumors, due to the inevitable depth-dependent decline of laser intensity. Radiotherapy has traditionally been one of the most common and effective anti-tumor methods by using ionizing radiation to destroy tumors, primarily through the generation of oxygen radicals. Although radiotherapy attacks crucial biomolecules (*e.g.*, DNA) inside cancer cells with no depth restriction,^94,95^ the non-specific nature and hypoxia-associated radioresistance restrict its clinic applications. Fortunately, some successful examples of combinatorial phototherapy and radiotherapy have been reported to overcome these restrictions.^47,96–98^

Since PDT and ionizing radiation aim at different therapeutic targets, their synergism in killing cells may produce a better therapeutic response. Evidence of combinatorial PDT and radiotherapy has been shown in some investigations, in which ionizing radiation is utilized to induce deeper PDT with diminished oxygen dependence.^99,100^ At the same time, PDT can also shorten the exposure time or reduce the radiation dose. For the combination of PTT and radiotherapy, PTT-induced hyperthermia caused not only irreversible damage to tumor tissue, but also increased intratumoral blood flow and oxygenation, thereby ameliorating the hypoxic condition in TME to facilitate cancer radiotherapy.^101^ Recently, various nanomaterials have been developed to improve PTT or radiotherapy.^101–104^ Take high-Z metal ions as an example, high-Z metal ions can strongly absorb, scatter, and re-emit radiation energy, then generate extra singlet oxygen to effectively concentrate a more significant local radiation within the tumor, thus offsetting radiotoxicity to surrounding normal tissues.^105^

**Fig. 6:**
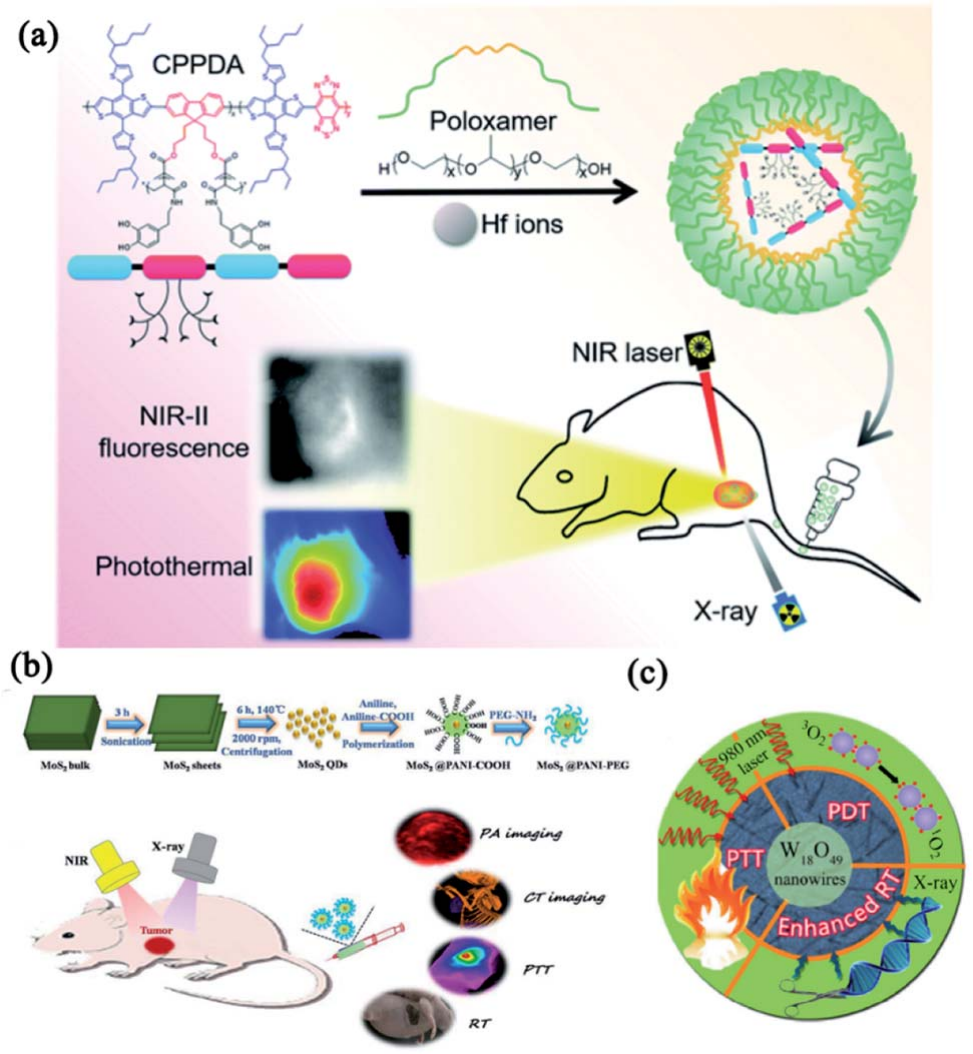
Combinatorial phototherapy and radiotherapy for enhanced cancer treatments. (a) Schematic illustration of the synthesis route of CPPDA-Hf@poloxamer for combinatorial therapy of PTT and radiotherapy. Reprinted with permission from Dai *et al.* (2021).^106^ (b) Synthesis of MoS_2_@PANI–PEG nanoparticles and their application in enhanced anti-tumor efficacy with the assistance of high Z element. Reprinted with permission from Li *et al.* (2016).^107^ (c) Schematic illustration of combinatorial effects between W_18_O_49_-PVP nanowire-mediated radiotherapy and PTT/PDT. Reprinted with permission from Bu *et al.* (2015).^108^

Dai's group designed a metal-polyphenolic framework (CPPDA-Hf) coated with an amphiphilic polymer (poloxamer) to construct a multifunctional nanomedicine (CPPDA-Hf@poloxamer) with combinatorial PTT and radiotherapy (Fig. 6a).^106^ In this system, the semiconducting polymer modified with dopamine moieties (CPPDA) acted as PTAs with intensive absorption in the second near-infrared (NIR-II) window. Hafnium ions (Hf, high-Z metal ions) were chelated with the polyphenol groups in dopamine (CPPDA-Hf). The CPPDA-Hf@poloxamer exhibited excellent synergism between PTT and radiotherapy, and acquired an amazing antitumor effect with the radio-sensitization effect of Hf ions under combined irradiation of 1064 nm laser and X-rays. Similarly, Li and coworkers developed a versatile nanomaterial based on MoS_2_ quantum dot@polyaniline inorganic–organic nanohybrids (MoS_2_@PANI–PEG).^107^ As shown in Fig. 6b, the nanohybrid not only exhibited imaging capacities of PAI and CT, but also performed efficient radiotherapy and PTT which remarkably improved the anti-tumor efficacy. Bu and co-workers fabricated a multifunctional nanoplatform for CT-guided combinatorial therapy.^108^ In their work, ultrathin polyvinyl-pyrrolidone (PVP)-decorated tungsten oxide (W_18_O_49_) nanowires (W_18_O_49_-PVP nanowires) could induce extensive heat and singlet oxygen-mediated damage to cancer cells when irradiated by NIR laser (Fig. 6c). These nanowires simultaneously functioned as radiation intensifying agents that enhance irradiative energy deposition locally and selectively during radiotherapy. The satisfying combinatorial effects between W_18_O_49_-PVP nanowire-mediated radiotherapy and PTT/PDT provided a potential application due to seamless integrating outcomes and imaging guidance.

### 2.4 Combination of phototherapy with immunotherapy

The tumor remains mortal and almost uncontrollable because of its highly invasive and metastatic nature. Although various phototherapeutic strategies have been developed to simultaneously inhibit cancer development and metastasis, the outcomes are still highly unsatisfactory.^109,110^ A promising paradigm for cancer treatment should have the total capacity to affect both primary tumor and any remaining tumor mass, like metastases. In recent years, immunotherapy has shown great potential in treating both primary and metastatic tumors.^111–115^ More importantly, phototherapy can lead to apoptotic immunogenic cell death (ICD),^116^ which not only directly ablate the tumor mass but also trigger a strong immune response. It is now accepted that the primary cancer cell destruction induced by phototherapy could cause the damage of plasma membrane, induce strong inflammatory responses, and generate tumor-associated antigens, including tumor-associated antigens, heat shock proteins (HSPs), and other danger-associated molecular patterns (DAMPs).^117^ However, the tumor fragments produced by phototherapy through activating tumor-specific T cells have exhibited unsatisfactory therapeutic efficacy in metastatic tumors since the mechanisms of immune escape were developed by tumors.^118^ Therefore, a combination of photo-immunotherapy could be a desirable approach to improve the antitumor effect.

**Fig. 7:**
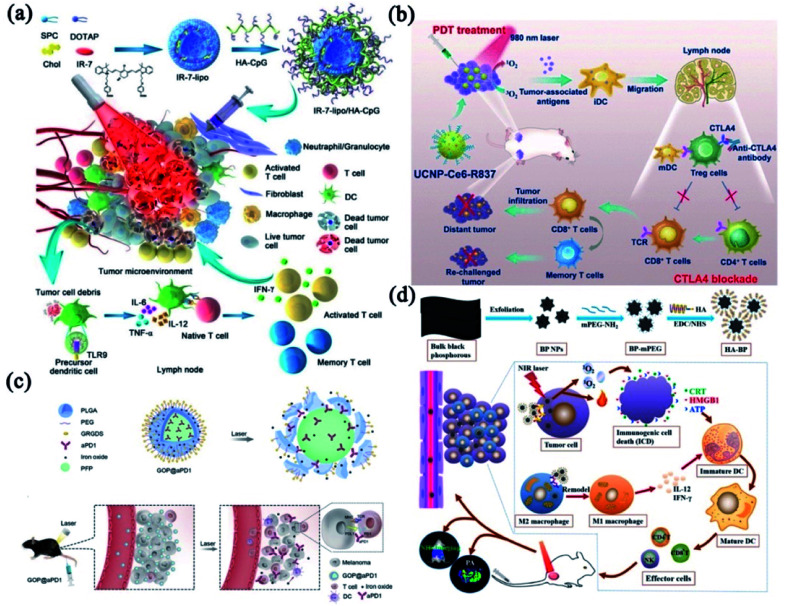
The combinatorial phototherapy and immunotherapy. (a) A schematic drawing of the synthesis and proposed working mechanism of IR-7-lipo/HA-CpG vaccine. Reprinted with permission from Gong *et al.* (2018).^119^ (b) Schematic showing the combinatorial PDT and CTLA4 blockade strategy. Reprinted with permission from Liu *et al.* (2017).^122^ (c) A schematic drawing of the proposed working mechanism and *in vivo* behavior of the GOP@aPD1 NPs. Reprinted with permission from Yang *et al.* (2019).^125^ (d) Synthesis strategy of HA-BP nanoparticles and *in vivo* antitumor immune responses. Reprinted with permission from Zhang *et al.* (2021).^126^

Combining PTT with immunotherapy would be an essential and effective strategy to augment the anti-tumor immune responses. Gong's group presented an endogenous vaccine based on fluorophore-loaded liposomes (IR-7-lipo) coated with a multivalent immunoadjuvant (HA-CpG) (Fig. 7a).^119^ Their results indicated that the PDT effect of IR-7-lipo/HA-CpG vaccine amplified the T-cell-mediated immune responses which played a vital role in controlling malignant diseases. It is worth noting that PDT is a more mature phototherapeutic strategy than PTT to combine with immunological adjuvants.^120,121^ Immune checkpoint blockades (ICBs) can be employed to strengthen the immune responses induced by PDT. As shown in Fig. 7b, Liu and co-workers designed an immune-stimulating UCNP-based PDT strategy combined with CTLA-4 blockade, which effectively promoted the PDT-induced immune responses, especially the immunological memory effect.^122^

Despite the fact that ICBs demonstrated the paramount importance of immunoregulatory in treating cancer, their clinical efficacy still needs to be improved.^123,124^ Antibody-directed delivery system with tumor-targeting functions could ensure the controllable release of phototherapeutic agents and ICBs to improve tumors immunotherapy. The immunogenic GOP@αPD1-based PDT^125^ particularly sensitized the tumor to αPD-1. In addition, the GOP@αPD1 NPs were modified with GRGDS peptides that could promptly identify tumors to reduce the potential off-target effect (Fig. 7c). Zhang *et al.* designed HA-BP nanoparticles modified by hyaluronic acid (HA) (Fig. 7d).^126^ HA modification not only enhanced CD44 receptor-mediated endocytosis of BP nanoparticles (targeting specificity), but also remodeled the phenotype of tumor-associated macrophage to significantly improve immunotherapeutic effect. Particularly, some treatments integrated phototherapy and chemo-immunotherapy have also been studied from a broader therapeutic perspective, the addition of chemotherapy contributes extra power to combat cancer.^25,127^ In conclusion, phototherapy-based immunotherapy has shown promising pre-clinical results in various tumor models owing to its exceptional advantages, such as specific antitumor immune responses and long-term immunological memory.^128,129^

### 2.5 Breakthrough of phototherapy with other therapies

The combination of phototherapy and gene therapy shows great potential to improve anti-tumor activity.^130–133^ The notable limitations of gene therapy, such as fast enzymatic degradation and low intracellular uptake rate *in vivo*, could be overcome by phototherapy. Nano-integrated strategy for synergetic tumor starvation and phototherapy also provides a feasible sensitization to cancer therapy, such as the interruption of blood supply^134^ and enzyme-mediated energy metabolism.^135,136^ The strategy that interrupts blood supply mainly includes anti-angiogenic strategies through blocking the function of angiogenic factors and obstructive thrombosis through transporting thrombin.^137,138^ Phototherapy combined with enzyme inhibitors can weaken the energy metabolism to cut off the energy supply, such as silencing the pyruvate kinase M2 (PKM2) in tumor cells.^135^ Tumor starvation therapy aims to block the blood supply, deplete glucose/oxygen and other critical nutrients of tumors for combinatorial effect, which renders anti-metabolic mechanism to sensitizing phototherapy. Additionally, high tumor interstitial pressure (TIP) leads to the unsatisfactory delivery efficiency of therapeutic agents.^139^ Herein, many efforts have been committed to reducing tumor interstitial fluid pressure (TIFP) *via* enzymolysis. For instance, we invented pyroelectric catalysis-based “nano-lymphatic” to decompose the tumor interstitial fluid, and reduce TIFP *via* pyroelectric catalysis-based water splitting, resulting in a smart combination of tumor-penetrating therapeutic strategy and photothermal therapy.^140^ Living photosynthetic bacteria (PSB) have been utilized as hypoxia-targeted carriers and PTAs for tumor therapy with hypoxia-targeting properties due to their near-infrared chemotaxis and physiological characteristics as facultative aerobes.^141^ Antibody-directed phototherapy (ADP)^142^ has been developed by conjugating a phototherapeutic agent to an antibody like ADCs (antibody–drug conjugates) do. With the assistance of antibodies, ADP can increase specificity and improve drug pharmacokinetics of phototherapeutic agents, thus showing more powerful *in vivo* performance than the free PTAs or photosensitizer. ADP is easy to prepare and with excellent targeting functions. However, ADP has some flaws, such as being easy to aggregate, poor pharmacokinetics, and loss of immuno-reactivity. Moreover, ADP barely integrates multiple therapeutic approaches. In comparison, nanotechnology-based phototherapy can combine the antibody to the surface and realize similar targeting functions. Not only that, nanotechnology can integrate different therapeutic strategies for synergistic effects, and the nanocarriers also protect the therapeutics from being degraded in the blood.

## 3. Conclusions and outlook

This review mainly discussed nanotechnology-based combinatorial phototherapy and its pros and cons in cancer treatments. Although PTT/PDT has non-invasive, controllable and accurate characteristics, phototherapy alone is hard to satisfy the needs for cancer treatments. A combination of PTT and PDT can minimize dose-dependent side effects during cancer treatments.^143^ Thanks to the fast development of nanotechnology, phototherapy can be integrated with other therapeutic approaches, especially chemotherapy, radiotherapy and immunotherapy, to remedy the defects and simultaneously overcome the intrinsic drawback of each monotherapy. Many preclinical works have demonstrated that phototherapy can facilitate intracellular translocation of anti-tumor drugs for enhanced chemotherapy with accurately controlled release properties.^144^ The MDR can be reversed through modulating the related efflux pumps and molecular signaling pathways with phototherapy. Size-changeable nanostructures with phototherapeutic effects are frequently employed to realize deep tissue penetration to improve delivery efficiency.^145,146^ Phototherapy exhibits satisfactory therapeutic effect to alleviate radioactive damage induced by non-specific radiotherapy and overcome hypoxia-associated radio-resistance. Non-negligible tumor metastasis and recurrence make cancer remain to be mortal and almost uncontrollable. Combining phototherapeutic agents with immunological adjuvants is an essential and effective strategy to augment the anti-tumor immune responses. Antibody-based phototherapy with a tumor-targeted strategy remedies the defective clinical efficacy. Additionally, combinatorial gene therapy/phototherapy strategies and other intelligent therapeutic systems are also discussed.

The past 30 years have witnessed the clinical attempts of PDT and PTT as approved or experimental treatment options for several solid tumor, but with limited clinical translation to date. The inherent drawbacks, mainly referring to the adverse events (AEs) associated with PDT/PTT, the limited penetration and non-negligible tumor recurrence, also restricted their widespread clinical use outside of certain dermatological indications.^147^ However, recognizing the potential of phototherapy, ongoing clinical trials, as registered on http://www.clinicaltrials.gov, focus on evaluating the safety, feasibility and efficacy of a variety of agents for PDT and PTT for numerous types of cancer and increase utilization in the clinic. In addition, preclinical studies are actively investigating approaches to overcome the obstacles encountered in clinical trials. Next-generation and nanoscale photosensitizing agents seem to have excellent preclinical results, especially the advanced targeting and activation features of agents, but it is undeniable that the complex modification might lead to manufacturing complexities that impede their clinical translation.^83^ Nevertheless, the building of multifunctional nanocarriers and the enlargement of indications for clinical use remain future development trends. Further studies are needed to show the applicability of advanced discoveries. Except for being an individual and powerful treatment, PDT and PTT are also expected to be part of a multimodal approach to cross the biological barrier and sensitize other therapies. In concision, phototherapy may offer clinically valuable therapeutic advantages in cancer treatment and considerable room exists for the clinical expansion of new PDT and PTT platforms.

## Conflicts of interest

There are no conflicts to declare.

## Supplementary Material
